# A Novel Therapy With a One-Month Ultrashort Regimen to Halt Progression From Latent Infection to Active Tuberculosis Among Close Contacts (The TB‑YOUTH Study): Protocol for a Cluster Randomized Controlled Trial

**DOI:** 10.2196/89584

**Published:** 2026-06-05

**Authors:** Yixuan Yang, Feiran Zhou, Yanfei Ren, Fudong Li, Qingluan Yang, Yuying He, Xiaoling Wei, Xing Wang, Jianfeng Luo, Feng Sun, Lingyun Shao, Jinlan Li, Qiaoling Ruan, Wenhong Zhang

**Affiliations:** 1 Department of Infectious Diseases, Shanghai Key Laboratory of Infectious Diseases and Biosafety Emergency Response, National Medical Center for Infectious Diseases, Huashan Hospital, Fudan University, No.12 Middle Wulumuqi Road, Shanghai, 200040, China, (8621)52888123; 2 Clinical Research Department, Shanghai Sci-Tech Inno Center for Infectious & Immunity, Shanghai, Shanghai, China; 3 Institute for Tuberculosis Control, Guizhou Center for Disease Control and Prevention, Guiyang, Guizhou, China; 4 Dalla Lana School of Public Health, University of Toronto, Toronto, ON, Canada; 5 Department of Biostatistics, School of Public Health, Fudan University, Shanghai, Shanghai, China; 6 See Acknowledgments

**Keywords:** mycobacteria tuberculosis, tuberculosis preventive treatment, TPT, close contacts, adolescent tuberculosis, clinical trial

## Abstract

**Background:**

Close contacts of individuals with active pulmonary tuberculosis (TB) face an elevated risk of TB acquisition, necessitating systematic screening for latent TB infection and subsequent TB preventive treatment (TPT). Major TPT regimens require ≥3 months of drug exposure and demonstrate suboptimal safety profiles, significantly compromising treatment completion rates. Therefore, the development of shorter, safer chemoprophylaxis strategies represents a critical need in global TB control. Among close contacts, school-aged children and adolescents constitute the most vulnerable demographic subgroup, warranting prioritized intervention efforts.

**Objective:**

The primary objective of this study is to demonstrate noninferiority of an ultrashort, 1-month TPT regimen of isoniazid plus rifapentine, administered 3 times a week (1H_3_P_3_) compared with the standard 3-month daily isoniazid plus rifampicin (3HR) regimen in preventing active TB, as measured by the 24-month cumulative incidence of active TB following randomization.

**Methods:**

An investigator-initiated, prospective, multicenter, open-label, noninferiority, cluster-randomized controlled clinical trial is being implemented under the auspices of the national TB control program in China. Close contacts of school pulmonary TB index cases, regardless of diagnostic type, are actively screened for symptoms using interferon-gamma release assays, chest imaging, and sputum molecular diagnostic testing to detect TB infection and exclude active TB. Eligible latent TB infection cases will be randomized in a 1:1 cluster ratio to receive either the standard 3HR regimen or the novel ultrashort 1H_3_P_3_ regimen for TPT, with subsequent follow-up for up to 2 years to assess disease progression. The primary composite end point includes microbiologically confirmed TB (sputum culture or molecular diagnostic testing) or clinically diagnosed TB. With 80% power to detect noninferiority (20% margin), the study requires 1760 participants per arm, accounting for cluster design effects.

**Results:**

Recruitment started in September 2023. By the end of December 2025, a total of 2478 participants, comprising 627 index cases, had been enrolled, and recruitment is estimated to continue until September 2026. Data analysis will commence after the 2-year follow-up period, and the results are expected to be published by March 2029.

**Conclusions:**

This cluster randomized controlled trial aims to establish the noninferiority of a novel, ultrashort 1H_3_P_3_ regimen compared to the standard 3-month 3HR regimen for preventing active TB in infected school contacts. If successful, this well-tolerated 1-month regimen could significantly improve treatment completion and optimize preventive therapy delivery in high-transmission congregate settings, thereby contributing substantively to global TB control efforts.

## Introduction

Latent tuberculosis infection (LTBI) refers to a state of persistent immune response to stimulation by *Mycobacterium tuberculosis* antigens without evidence of clinically manifest active tuberculosis (TB) and without bacteriological confirmation of active disease [1]. This immunological equilibrium can shift under conditions of waning host immunity, leading to active TB disease. On average, epidemiological data indicate a 5% to 10% lifetime risk of developing active TB, usually within 5 years after initial infection [1,2]. The incidence increases in high-risk populations, such as recent close contacts and immunocompromised individuals [3,4]. Screening and treating TB infection in the latent state are essential components of the World Health Organization End TB Strategy to prevent active disease [5]. Emerging transmission models suggest that a large proportion of transmission at the population level may occur outside the household [6]. This is particularly relevant in educational institutions, where congregate settings amplify transmission risk. For example, school-acquired infections constitute >50% of TB in adolescents (aged 15 to 19 years) [7].

TB preventive treatment (TPT) has been shown to effectively prevent adolescents with LTBI from progressing to active disease [8,9]. A TPT project conducted in Tibetan schools in India showed excellent efficacy, tolerability, and treatment adherence with the currently recommended 3-month daily isoniazid plus rifampicin (3HR) preventive therapy [10]. However, the same 3HR regimen for TPT has only resulted in low adherence and completion rates in practice in China, primarily attributed to its prolonged duration and drug-related toxicity [11]. To address these limitations, we propose a novel ultrashort 1-month TPT regimen of isoniazid and rifapentine, administered 3 times a week (1H_3_P_3_), through phased clinical development. In a pilot study, the regimen demonstrated improved safety and potentially superior efficacy compared to the 3-month isoniazid plus rifapentine regimen in patients with silicosis in Zhejiang province [12]. In particular, the treatment completion rate increased to >90%.

Therefore, we designed this trial to demonstrate the noninferiority of the ultrashort course of the 1H_3_P_3_ TPT regimen compared to the standard 3HR regimen in preventing active TB, as measured by the 24-month cumulative incidence of active TB following randomization. The findings will empirically define the clinical utility of accelerated TPT to support evidence-based clinical practice and policy development for TB elimination.

## Methods

### Study Design

This is a multicenter, prospective, cluster randomized, noninferiority clinical trial comparing the protective efficacy and safety of the 1H_3_P_3_ regimen with those of the 3HR regimen for TPT in adolescents and adults with LTBI. Prior to trial initiation, close contacts of pulmonary TB index cases within school environments will undergo active screening through the TB-YOUTH Screening program, a cross-sectional study (ClinicalTrials.gov NCT06033807), under the auspices of the National Tuberculosis Control Program in China. After excluding active TB, eligible LTBI participants will be grouped into clusters by their corresponding index cases. The clusters will then be randomized in a 1:1 ratio to receive either the 1H_3_P_3_ or the 3HR regimen for TPT. Medication adherence and adverse events (AEs) will be monitored at week 2, month 1, and month 2 for the 1H_3_P_3_ group, and at week 2 and then monthly until month 4 for the 3HR group after treatment initiation. Participants will undergo follow-up clinical assessments every 6 months for 2 years following randomization. The preventive efficacy of each study arm against active TB will be evaluated at the conclusion of the follow-up period.

### Description of Process

#### Identification of Index Cases and Contacts

The school contact screening cascade will be activated upon notification of active TB cases involving currently attending students or faculty members through the National Infectious Disease Surveillance System. Following verification by the local Centers for Disease Control and Prevention, comprehensive epidemiological and clinical data on the index case (eg, drug susceptibility profiles) will be systematically collected. Concurrently, TB screening will commence for school contacts of the index case. School contacts are defined in alignment with school TB prevention and control guidelines in China, which include teachers and students in the same class as the index case and students cohabiting in the same dormitory with the index case for at least 4 hours per week [13]. Operational definitions for screening algorithms are detailed in the TB-YOUTH Screening protocol.

#### Enrollment of LTBI Contacts

Contacts testing positive on QuantiFERON-TB Gold Plus (QIAGEN N V) will subsequently receive chest computed tomography (CT) or chest X-ray, as well as pooled sputum nucleic acid amplification testing (eg, GeneXpert MTB-RIF [Danaher Corp]) to exclude active TB. LTBI is defined as a positive interferon-gamma release assay result without evidence of active TB. Those with confirmed LTBI and aged ≥13 years are eligible for this trial. Study protocols will be thoroughly explained to eligible participants by site investigators, with written informed consent obtained prior to enrollment. Parental or guardian consent is mandatory for minors (aged <18 years). Participants meeting the inclusion criteria but declining enrollment in the TPT trial will be invited to join a supplemental nonintervention cohort. After providing separate informed consent, they will be followed for 24 months to monitor for the development of active TB. Further eligibility validations will require review of medical history and comprehensive laboratory evaluations to exclude contraindications to TPT. Textbox 1 shows the detailed inclusion and exclusion criteria.

Textbox 1. **Inclusion and exclusion criteria for the TB-YOUTH study.**

**Inclusion criteria**

- Aged ≥13 years and body weight ≥30 kg
- School-registered individuals, including the following:
  - Students currently attending junior high school, senior high school, or university
  - School staff members
- Close contacts of active pulmonary tuberculosis (TB) index cases (microbiologically confirmed or clinically diagnosed) within the school, defined as meeting both the following criteria:
  - Teachers or students sharing the same classroom or dormitory as the index case
  - Exposure history defined as prolonged sharing of an enclosed space (>4 hours total within 1 week) with the index case
- Confirmed latent TB infection (LTBI) status through screening
- Voluntary participation with a signed informed consent form (for adults aged ≥18 years)
- Parental or guardian consent with cosigned informed consent form (for minors aged 13 to 17 years)

**Exclusion criteria**

- Current active TB disease (clinically or bacteriologically confirmed)
- Documented isoniazid or rifampicin resistance in the corresponding *Mycobacterium tuberculosis* strain from the index case
- Self-reported use of rifamycins (eg, rifampicin or rifapentine) or isoniazid for >14 consecutive days within the past 2 years
- Prior completion of a full course of treatment for active TB or LTBI
- Hypersensitivity or intolerance to rifamycins (rifapentine or rifampicin) or isoniazid
- HIV-positive serostatus or diagnosis of AIDS
- History of viral hepatitis (eg, chronic hepatitis B or chronic hepatitis C) or liver cirrhosis
- Liver dysfunction (total bilirubin >2.5 mg/dL [43 μmol/L] or alanine aminotransferase/aspartate aminotransferase >2×upper limit of normal) or renal dysfunction
- Currently receiving immunosuppressive therapy or biological agents
- Hematologic disorders, with either platelet count <50×10^9^/L or white blood count <3.0×10^9^/L
- Other conditions deemed unsuitable for TB preventive treatment by investigators

### TPT Intervention

Eligible participants, clustered by epidemiologically linked index cases, will undergo 1:1 randomization through an interactive web response system into 2 arms: the intervention arm will receive a 12-dose 1H_3_P_3_ regimen, and the active control arm will receive a 90-dose 3HR regimen. Intervention discontinuation should be considered under the following circumstances: (1) at any time upon request by the participant and/or their guardian, (2) major protocol deviation, or (3) an AE deemed by the safety review committee to result in an unfavorable risk-benefit profile for the participant. Interruption of one or more drugs of the assigned regimen consecutively for ≥1 month will be considered permanent discontinuation. Table 1 shows the detailed dosage and administration protocol of study drugs in each regimen. Any dose adjustment is strictly prohibited and will be considered a protocol deviation. All medications will be provided free of charge. TPT other than the assigned intervention is prohibited during the study.

**Table 1.** Dosage and administration of study drugs for the 1H_3_P_3_ (1-month isoniazid plus rifapentine 3 times weekly) and 3HR (3-month daily isoniazid plus rifampicin) regimens.

Regimen and dosing categories (age and/or weight)	Dosage			Administration	Treatment course (months)
	Isoniazid	Rifapentine	Rifampicin		
1H_3_P_3_					
Weight 30-35 kg	300 mg	300 mg	N/A^a^	3 times weekly	1
Weight ≥35 kg	400 mg	450 mg	N/A	3 times weekly	1
3HR					
Age ≥18 years					
Weight <50 kg	300 mg	N/A	450 mg	Once daily	3
Weight ≥50 kg	300 mg	N/A	600 mg	Once daily	3
Age <18 years (weight-based dosing)	10 mg/kg^b^	N/A	10 mg/kg^c^	Once daily	3

a N/A: not applicable.

b Maximum dose of isoniazid=300 mg.

c Maximum dose of rifampicin=450 mg.

### Participant Monitoring and Support

All screened contacts will receive comprehensive TB education (eg, on-site education class, pamphlets, and digital resources) during initial and follow-up screenings. Participants enrolled in TPT will receive a standardized medication diary to log each administration (time, dose, and any AEs). Investigators will review entries prior to dispensing subsequent doses. A patient education booklet summarizing key TPT information (eg, frequently asked questions derived from the pilot study) will be distributed with the diary. Participants within clusters will be encouraged to select a peer coordinator to facilitate group medication adherence and foster a peer-supported compliance network.

### Follow-Up and Assessments

Participants in both study groups will undergo prospective monitoring comprising two phases: (1) on-treatment assessment (from enrollment to 1 month after treatment completion) and (2) posttreatment assessment (up to 24 months after randomization). On-treatment assessments will involve standardized clinical evaluations, including physical signs, laboratory testing, medication adherence (pill counts and self-reporting), and safety monitoring, and will be scheduled at week 2, month 1, and month 2 for the 1H_3_P_3_ arm, and at week 2, then monthly until month 4 for the 3HR group. On the basis of recommendations from the independent protocol review board, and to comprehensively evaluate the impact of interventions on quality of life, a protocol amendment was implemented after approximately 2300 participants had been enrolled. Following ethics committee approval, the EuroQol 5-Dimensions 3-level questionnaire (version 6.0, December 2018) was incorporated as an exploratory end point. This questionnaire will be administered at every on-treatment assessment for participants enrolled after the amendment’s effective date. Posttreatment assessment will focus on active TB surveillance as well as delayed AE monitoring and will occur every 6 months from randomization until study exit. At each visit, participants will receive a standardized symptom screening for active TB, including persistent cough (≥2 weeks), fever, night sweats, weight loss, hemoptysis, or other TB-associated symptoms. Participants with positive symptom screens will undergo confirmatory sputum culture and molecular diagnostic testing. The end point adjudication committee will determine the final active TB diagnosis per predefined criteria.

The schedule of this study is summarized in Table 2.

**Table 2.** SPIRIT (Standard Protocol Items: Recommendations for Interventional Trials) diagram showing enrollment, intervention, and assessment procedures.

	Enrollment	Allocation	Postallocation phase						Closeout
Time point^a^	–30 days	0 day	2 weeks	1 month	2 months	3 months	4 months	Every 6 months	24 months
Enrollment									
Eligibility screening	✓								
Informed consent	✓								
Medical history	✓								
Allocation		✓							
Interventions									
1-month isoniazid plus rifapentine 3 times weekly regimen		↔^b^	↔	↔					
3-month daily isoniazid plus rifampicin regimen		↔	↔	↔	↔	↔			
Assessments^c^									
Demographic characteristics and medical history	✓	✓							
Treatment adherence			✓	✓	✓	✓			
Safety monitoring			↔	↔	↔	↔	↔		
Tuberculosis surveillance		↔	↔	↔	↔	↔	↔	↔	↔

a This row displays specific time points relative to randomization. Acceptable time window: more or less than 7 days for the intervention period and more or less than 30 days for the postintervention phase.

b ↔: this arrow represents continuous interventions.

c On-treatment assessments and 1-month posttreatment assessments require in-person attendance, whereas postintervention assessments may be conducted in person or remotely.

### Study Objectives and End points

The primary objective is to demonstrate noninferiority of the ultrashort course of the 1H_3_P_3_ TPT regimen compared with the standard 3HR regimen in preventing active TB, as measured by the 24-month cumulative incidence of active TB following randomization. The secondary objectives include: (1) to compare safety, patient-reported quality of life, and treatment discontinuation rates attributable to AEs between the regimens; (2) to evaluate treatment adherence using protocol-defined completion criteria; (3) to assess acquired *M tuberculosis* drug resistance among participants who develop active TB after TPT; and (4) to establish a contemporary nonintervention cohort for exploratory comparison, delineating the natural progression to active TB among individuals with high-risk LTBI in whom active TB has been ruled out.

The primary end point is a composite of bacteriologically confirmed or clinically diagnosed active TB within 24 months after randomization. Bacteriologically confirmed active TB refers to *M tuberculosis* identification via ≥1 sputum culture and/or molecular assay (eg, GeneXpert MTB-RIF) with detectable bacterial load. Clinically diagnosed TB combines objective clinical symptoms (≥2 of the following: cough ≥2 weeks, fever, night sweats, weight loss >10%, or hemoptysis) and imaging findings highly suggestive of active TB (eg, cavity or nodular lesions on chest CT), after exclusion of alternative diagnoses. Secondary end points include severity-graded AEs, treatment discontinuation (defined as any permanent cessation prior to regimen completion), treatment completion (defined as completing ≥90% of scheduled doses within 110% of the planned treatment duration), all-cause mortality, and acquired drug resistance determined by drug susceptibility testing or targeted molecular assays of *M tuberculosis* isolates from active TB cases. Exploratory analysis will use the nonintervention cohort to establish the background incidence of active TB, against which the risk reduction of each TPT regimen can be calculated. Patient-reported outcomes (PROs) will be calculated using health state utility values and EuroQol visual analog scale scores.

### Randomization and Sample Size Calculation

Enrolled LTBI participants linked to the same index case will be defined as a cluster [14,15], with an assumed average cluster size of 4 participants. Cluster-level randomization will be triggered when ≥1 eligible participant is enrolled within a cluster. Randomization sequences will be pregenerated by the study statistician using SAS (version 9.4; SAS Institute Inc) and will not be accessible to any personnel involved in enrollment, assignment, or intervention delivery. Designated personnel will perform centralized randomization via the interactive web response system. Randomization results will be relayed to the respective site investigators. This is an open-label study, and all participants within the same cluster will receive identical group assignments. To address intercenter heterogeneity, a 2-stage randomization strategy will be applied: in the first stage, stratification by study site will balance baseline characteristics across centers; in the second stage, permuted block randomization (block size=4) within each site will ensure immediate group balance during participant enrollment.

On the basis of surveillance data from China’s National Tuberculosis Control Program, we estimate that the 2-year cumulative active TB incidence among LTBI participants will be 7% [16,17]. Both the 3HR and 1H_3_P_3_ regimens are assumed to have 70% protective efficacy (ie, reducing the 2-year cumulative incidence of active TB into 2.1%).

The intracluster correlation coefficient (ICC) was incorporated into calculations. A systematic review reported ICC values of 0.028 (range 0.0005‐0.21) for school-based clusters [15], while the National Institutes of Health Pragmatic Trials Collaboratory suggested a range of 0.01 to 0.05 [18]. Therefore, we adopted a conservative ICC estimate of 0.05 for this study.

Using these parameters and a prespecified noninferiority margin of a 20% relative reduction in protective efficacy, a total of 396 clusters (n=1584 participants) per treatment arm are required to achieve 80% power at a 1-sided *α*=.05. This sample size is calculated to satisfy the clinically meaningful noninferiority margin, accounting for the cluster randomized design (ICC=0.05, average cluster size=4). Accounting for a 10% attrition rate to mitigate the impact of loss to follow-up on statistical power and outcome validity, each arm will enroll 1760 participants.

### Data Collection and Management

The trial workflow adheres to the SPIRIT (Standard Protocol Items: Recommendations for Interventional Trials) statement [19]. Site investigators and staff from each center will undergo centralized training to standardize protocols for participant screening, intervention delivery, follow-up procedures, end point adjudication, and AE documentation and management. Each participant will be assigned a unique anonymized identifier, accessible only to authorized personnel for monitoring purposes. Data will be prospectively captured on paper-based case report forms (CRFs) by on-site investigators or clinical research coordinators, followed by single-entry migration into a customized electronic data capture system. The electronic data capture platform enforces role-based access control to ensure confidentiality. Data integrity audits will be conducted monthly by the data management team. Upon trial completion, the central coordinating office will execute database locks, after which only 2 primary investigators and 1 independent statistician will retain access to the final analytic dataset.

For the primary end point assessment, investigators will perform symptom screening (eg, cough, fever, night sweats, weight loss, and hemoptysis) and physical examination to identify suspected active TB cases at each visit. Participants meeting clinical criteria will undergo confirmatory testing, including chest imaging (preferably chest CT), sputum culture, and sputum molecular testing (eg, GeneXpert MTB-RIF). All suspected cases will undergo centralized adjudication by an independent TB expert panel to confirm the diagnosis.

For secondary end points assessment, AEs will be documented in a dedicated CRF section, capturing event type, onset date, severity (graded according to Division of AIDS criteria), presumed causality, management, and resolution status. Medication adherence will be tracked through participant interviews, drug diary review, and pill counts; discrepancies will be resolved by prioritizing measured residual drug quantities. Survival status will be verified at each visit. Postdropout mortality ascertained through external sources will trigger outcome reclassification from “loss to follow-up” to “deceased.” Drug resistance profiling via molecular assays will be performed exclusively in bacteriologically confirmed active TB cases.

### Statistical Analysis

Statistical analyses will be performed using SAS and R (R Foundation for Statistical Computing). Multiple imputation will be conducted to handle missing data, with additional inverse probability weighting applied for the nonintervention cohort to adjust for potential attrition bias. Continuous variables will be summarized as mean (SD) or median (IQR), as appropriate. Categorical variables will be summarized as counts and percentages.

The primary analysis will assess the noninferiority of the 1H_3_P_3_ regimen compared to the 3HR regimen in preventing active TB over the 24-month follow-up period, and the primary end point is the composite of bacteriologically confirmed or clinically diagnosed active TB. To account for the cluster randomized design and the inherent correlation within clusters, we will use a Cox proportional hazards regression model with shared frailty terms and exact likelihood estimation. Noninferiority of the 1H_3_P_3_ regimen relative to the 3HR regimen will be concluded if the analysis demonstrates that the 1H_3_P_3_ regimen does not experience a ≥20% relative reduction in protective efficacy for preventing active TB over the 24-month follow-up period. To enhance precision and address potential baseline imbalances despite randomization, the model will adjust for key prespecified covariates, such as age, sex, BMI, characteristics of the index case (smear or cavitation status), and cluster size. Outcomes will be assessed via 2 prespecified analyses with explicit definitions: the modified intention-to-treat population includes all randomized participants who received at least 1 dose of the study medication, analyzed by the assigned treatment group regardless of subsequent protocol deviations or treatment discontinuation; the per-protocol population includes all randomized participants who completed ≥90% of scheduled doses within 110% of the planned treatment duration without major protocol deviations (including use of prohibited concomitant anti-TB medications, enrollment despite exclusion criteria, or unblinding to outcome assessors). Prespecified subgroup analyses will examine treatment effect heterogeneity by age (13-17 years vs ≥18 years) and index case features (smear or cavitation status), tested through interaction terms in Cox models (interaction *P*<.10).

For secondary end points, all AEs, serious AEs (SAEs), and treatment discontinuations due to AEs will be documented. Group differences in AE proportions will be analyzed using the chi-square test or Fisher exact test (2-sided *α*=.05). Treatment completion is defined as consuming ≥90% of planned doses within 110% of the planned treatment duration (≥11 doses within 6 weeks for the 1H_3_P_3_ group and ≥81 doses within 16 weeks for the 3HR group). The 2-year cumulative incidence of active TB in the nonintervention cohort will be calculated using the Kaplan-Meier method, with 95% CI estimated. The protective efficacy of the 1H_3_P_3_ and 3HR regimens relative to the nonintervention cohort will be quantified as the risk reduction rate: 1–(intervention group incidence/nonintervention group incidence). The Fisher exact test will be used to compare incidence differences among the 3 cohorts.

All analyses concerning the PRO end point will be explicitly restricted to the subgroup of participants who complete the EuroQol 5-Dimensions 3-level questionnaire. The baseline characteristics of this PRO subgroup will be compared between the 2 arms to confirm that randomization balance was maintained. Any interpretation of PRO findings will explicitly acknowledge their exploratory nature and the fact that they are derived from a subset of the total study population. Trends in PRO metrics over time will be visualized using line graphs, with mean values and 95% CIs plotted to illustrate group-level changes. A mixed-effects model for repeated measures will be used to compare PRO differences between the 1H_3_P_3_ and 3HR groups across time points, with fixed effects including treatment group, assessment time point, treatment group×time point interaction, age, sex, BMI, and index case cavitation status, and a random participant-specific intercept to account for within-subject correlation from repeated assessments.

### AE Management

AE severity will be graded per the standardized Division of AIDS table (version 2.1) [20]. A safety review committee composed of 3 independent infectious disease experts will oversee safety monitoring and conduct periodic reviews of safety data. All AEs will be systematically assessed for their causal relationship to study medications and categorized as related (adverse drug reaction), unrelated, or indeterminate (insufficient evidence for classification). AEs identified during the study will be promptly documented in the dedicated AE module of the CRF. SAEs necessitate immediate notification: study investigators or site personnel must report SAEs to the local ethics committee and study safety board within 24 hours of detection, followed by comprehensive documentation including severity, causality, and potential corrective actions.

### Quality Assurance

All participating sites are regionally designated medical centers for TB, staffed by experienced clinical teams. Prior to study initiation, all personnel will complete mandatory protocol training, including certification in intervention delivery, data collection standards, and compliance monitoring. Dedicated project managers will provide oversight of drug accountability procedures and on-site auditing to ensure protocol adherence and perform trial conduct monitoring quarterly.

Medication adherence is one of the key assessments for this study. Thus, adherence monitoring uses a 3-strategy verification framework: direct participant reporting during follow-up visits, cross-checking of self-administered medication diaries, and quantitative pill counts of returned drug containers. Within each cluster, peer coordinators will conduct weekly adherence reminders and provide real-time troubleshooting for medication-related challenges.

### Ethical Considerations

Participants will be identified and recruited through active screening of close contacts associated with school-based TB index cases. Prior to enrollment, informed consent will be mandatorily obtained, ensuring adequate time for eligible participants and, if needed, their guardians to review study details and voluntarily decide on participation. For individuals under the age of 18 years or those lacking decision-making capacity, written consent will be obtained from their guardians or representatives as well. To standardize ethical practices, the research team will conduct targeted training sessions for all participating health personnel, covering protocols for ethical consent procedures and strategies to minimize participant stress. All staff are required to deliver compassionate support throughout the study and provide psychological counseling if needed. For those who experience harm from study participation, free treatment and appropriate financial compensation will be offered. The study has been reviewed and approved by the ethics committee of Huashan Hospital, Fudan University (2023M-020), and will undergo annual continuing review. Protocol amendments require rereview and approval before implementation. Measures will be taken to protect participant privacy and to ensure confidentiality throughout the study. First, all identifiable personal information (eg, name and phone number) will be separated from other study data once collected, then replaced with unique anonymized identifiers. Second, study data will be stored on an encrypted server that is accessible only to core staff. Third, when obtaining informed consent, participants will be explicitly informed about data anonymization, secured storage, the scope of data use, and the prohibition against data sharing with third parties without participants’ permission. Last, participants will be informed of their rights to withdraw from the study at any time and to request deletion of their data. No identifiable features will appear in any published materials, and all reported results will be aggregated and deidentified.

## Results

Recruitment started in September 2023. By the end of December 2025, a total of 2478 participants, comprising 627 index cases, had been enrolled, and the recruitment is estimated to continue until September 2026. Data analysis will commence after the 2-year follow-up period, and the results are expected to be published by March 2029.

## Discussion

A systematic review highlighted an inverse association between TPT duration and completion rates, emphasizing that shortening treatment duration could significantly improve adherence and completion rates (odds ratio 1.54, 95% CI 1.04-2.29) [21]. Although high treatment completion rates are critical for achieving optimal protective efficacy, prior trials of TPT reported completion rates ranging from 60% to 80% [21-23]. Although the 12-week weekly rifapentine plus isoniazid regimen achieved 95.7% completion in people living with HIV [24], this rate substantially declined to 82.1% in the general population [23]. Moreover, safety concerns regarding the increased frequency of systemic drug reactions also began to surface during large-scale implementation [25]. Although current guidelines have included the 1-month daily rifapentine plus isoniazid regimen [1], its applicability is limited by the high pill burden. The dosage of rifapentine in our innovative intermittent-dosing 1H_3_P_3_ regimen has been reduced, potentially offering superior acceptability and cost-effectiveness. Compared with the daily rifapentine-based regimen, intermittent administration of rifapentine is preferable due to its long-acting nature. When previously validated in patients with silicosis, the 1H_3_P_3_ regimen demonstrated excellent tolerability (systemic reaction rate=0.8%; grade 3-4 AE=0.4%) and superior completion rates (92.0%). Notably, despite a >30% reduction in total drug exposure compared to a 1-month daily rifapentine plus isoniazid regimen, this regimen maintained protective efficacy without compromise [12].

School-based recruitment was strategically selected for 3 reasons. First, adolescent students resemble household contacts in their heightened vulnerability to TB infection; their prolonged congregate living in enclosed environments amplifies transmission risks, necessitating urgent LTBI screening and intervention [26,27]. Second, although students constitute a priority population for TB control in China, many LTBI cases remain undiagnosed and untreated [28]. This study addresses this critical gap. Third, the organized school environment facilitates coordinated medication administration and follow-up, ensuring rigorous protocol implementation. Integrated with the ongoing TB-YOUTH Screening initiative, our trial aims to establish a comprehensive school TB control model.

China has prioritized school TB control, with provincial Centers for Disease Control and Prevention in high-burden areas implementing expanded screening programs to detect TB cases among students [29]. Despite operational challenges, this study aligns with national efforts and receives multisectoral support from education authorities and social media platforms. As evidenced by the “zero TB” initiative in India, community-engaged strategies can achieve excellent TPT acceptance and adherence [10].

Cluster randomization was adopted for several reasons. First, participants in close-contact settings (eg, classmates and dormitory residents) have prolonged shared exposure, increasing the risk of intracluster interference. Second, distinct intervention protocols between groups (daily vs intermittent dosing) could cause confusion if implemented individually. Cluster-level administration allows mutual adherence reminders among participants, potentially enhancing adherence. Teachers and other school staff meeting close-contact criteria will be coenrolled in the same cluster as students to maintain intervention homogeneity.

We adopted a 20% relative reduction in protective efficacy as the noninferiority criterion, which is clinically justified and aligns with the core objective of evaluating whether the ultrashort 1H_3_P_3_ regimen retains adequate protective efficacy compared to the standard 3HR regimen. Historical data support this threshold: the 3HR regimen reduces TB incidence from 7% to 2.1% (70% protective efficacy), and a 20% relative reduction in protective efficacy is clinically acceptable for the 1H_3_P_3_ regimen. Although noninferiority is the primary focus, the regimen’s ultrashort duration and favorable safety profile could offer substantial practical benefits for individuals with LTBI.

The study also has some potential limitations. First, cluster randomization requires a larger sample size, posing challenges in resource-limited settings. Second, in complicated school settings, close contacts may extend beyond predefined clusters (eg, cross-classroom interactions), making cluster assignment more complicated. To mitigate this issue, clusters will be defined by the earliest identified index case, even when multiple potential sources of infection are present. Third, the school-based population may limit generalizability. In the original design, only students with LTBI were included. To improve demographic representation, the revised criteria were broadened to include teachers and staff. Nevertheless, adherence patterns in this study population may still not fully be representative of those of the general population. Pragmatic implementation studies will be needed to validate real-world applicability.

In conclusion, this cluster randomized noninferiority trial evaluates a novel 1H_3_P_3_ regimen for TB prevention among adolescents and adults with LTBI. If successful, this ultrashort, well-tolerated regimen could optimize TPT delivery, particularly in congregate settings. When integrated with active screening, our findings may inform scalable TB control strategies, advancing progress toward global TB elimination goals.

## Supplementary material

10.2196/89584 Checklist 1 SPIRIT 2025 checklist for randomized trials.

## Abbreviations

1H_3_P_3_: 1-month isoniazid plus rifapentine, administered 3 times weekly
3HR: 3-month daily isoniazid plus rifampicin
AE: adverse event
CRF: case report form
CT: computed tomography
ICC: intracluster correlation coefficient
LTBI: latent tuberculosis infection
PRO: patient-reported outcome
SAE: serious adverse event
SPIRIT: Standard Protocol Items: Recommendations for Interventional Trials
TB: tuberculosis
TPT: tuberculosis preventive treatment

## References

[1] (2024). WHO consolidated guidelines on tuberculosis: module 1: prevention - tuberculosis preventive treatment, second edition. World Health Organization.

[2] Martinez L, Cords O, Horsburgh CR, Andrews JR, Pediatric TB Contact Studies Consortium (2020). The risk of tuberculosis in children after close exposure: a systematic review and individual-participant meta-analysis. Lancet.

[3] Warner DF, Barczak AK, Gutierrez MG, Mizrahi V (2025). Mycobacterium tuberculosis biology, pathogenicity and interaction with the host. Nat Rev Microbiol.

[4] Trajman A, Campbell JR, Kunor T (2025). Tuberculosis. Lancet.

[5] (2022). Implementing the end TB strategy: the essentials, 2022 update. World Health Organization.

[6] Martinez L, Shen Y, Mupere E, Kizza A, Hill PC, Whalen CC (2017). Transmission of mycobacterium tuberculosis in households and the community: a systematic review and meta-analysis. Am J Epidemiol.

[7] Andrews JR, Morrow C, Walensky RP, Wood R (2014). Integrating social contact and environmental data in evaluating tuberculosis transmission in a South African township. J Infect Dis.

[8] Snow KJ, Cruz AT, Seddon JA (2020). Adolescent tuberculosis. Lancet Child Adolesc Health.

[9] Martinez L, Seddon JA, Horsburgh CR, Lange C, Mandalakas AM, TB Contact Studies Consortium (2024). Effectiveness of preventive treatment among different age groups and Mycobacterium tuberculosis infection status: a systematic review and individual-participant data meta-analysis of contact tracing studies. Lancet Respir Med.

[10] Dorjee K, Topgyal S, Dorjee C (2019). High prevalence of active and latent tuberculosis in children and adolescents in Tibetan schools in India: the Zero TB Kids Initiative in Tibetan refugee children. Clin Infect Dis.

[11] Peng L, Zhou Y, Wang M (2025). Acceptance of preventive therapy for latent tuberculosis infection in Chinese children and adolescents: a systematic review and meta-analysis. Sci Rep.

[12] Ruan QL, Yang QL, Ma CL (2025). Efficacy and safety of a novel short course rifapentine and isoniazid regimen for the preventive treatment of tuberculosis in Chinese silicosis patients: a pilot study (SCRIPT-TB). Emerg Microbes Infect.

[13] Xia Y, Chen H, Zhang C, Zhao Y, Cheng J, Zhang H (2021). Guidelines for the prevention and control of tuberculosis in schools: recommendations from China CDC. China CDC Wkly.

[14] Parker K, Nunns MP, Xiao Z, Ford T, Ukoumunne OC (2021). Characteristics and practices of school-based cluster randomised controlled trials for improving health outcomes in pupils in the UK: a systematic review protocol. BMJ Open.

[15] Parker K, Nunns M, Xiao Z, Ford T, Ukoumunne OC (2021). Characteristics and practices of school-based cluster randomised controlled trials for improving health outcomes in pupils in the United Kingdom: a methodological systematic review. BMC Med Res Methodol.

[16] Gao L, Bai L, Liu J (2016). Annual risk of tuberculosis infection in rural China: a population-based prospective study. Eur Respir J.

[17] Gao L, Li X, Liu J (2017). Incidence of active tuberculosis in individuals with latent tuberculosis infection in rural China: follow-up results of a population-based, multicentre, prospective cohort study. Lancet Infect Dis.

[18] Analysis plan: section 2: intraclass correlation. Rethinking Clinical Trials.

[19] Chan AW, Boutron I, Hopewell S (2025). SPIRIT 2025 statement: updated guideline for protocols of randomized trials. Nat Med.

[20] (2017). Division of AIDS (DAIDS) table for grading the severity of adult and pediatric adverse events. National Institute of Allergy and Infectious Diseases, National Institutes of Health, US Department of Health and Human Services.

[21] Sandgren A, Vonk Noordegraaf-Schouten M, van Kessel F, Stuurman A, Oordt-Speets A, van der Werf MJ (2016). Initiation and completion rates for latent tuberculosis infection treatment: a systematic review. BMC Infect Dis.

[22] Menzies D, Adjobimey M, Ruslami R (2018). Four months of rifampin or nine months of isoniazid for latent tuberculosis in adults. N Engl J Med.

[23] Sterling TR, Villarino ME, Borisov AS (2011). Three months of rifapentine and isoniazid for latent tuberculosis infection. N Engl J Med.

[24] Martinson NA, Barnes GL, Moulton LH (2011). New regimens to prevent tuberculosis in adults with HIV infection. N Engl J Med.

[25] Bhargava A (2024). The 3 HP regimen for tuberculosis preventive treatment: safety, dosage and related concerns during its large-scale implementation in countries like India. Lancet Reg Health Southeast Asia.

[26] Moscibrodzki P, Enane LA, Hoddinott G (2021). The impact of tuberculosis on the well-being of adolescents and young adults. Pathogens.

[27] Choi Y, Park SJ, An HS (2025). Epidemiological analysis of tuberculosis transmission, risk factors, and subclinical tuberculosis management in a high school outbreak, South Korea. Open Forum Infect Dis.

[28] Bunyasi EW, Geldenhuys H, Mulenga H (2019). Temporal trends in the prevalence of *Mycobacterium tuberculosis* infection in South African adolescents. Int J Tuberc Lung Dis.

[29] (2024). Towards a TB-free India, village by village. World Health Organization.

